# The quality of reporting in cluster randomised crossover trials: proposal for reporting items and an assessment of reporting quality

**DOI:** 10.1186/s13063-016-1685-6

**Published:** 2016-12-06

**Authors:** Sarah J. Arnup, Andrew B. Forbes, Brennan C. Kahan, Katy E. Morgan, Joanne E. McKenzie

**Affiliations:** 1 School of Public Health and Preventive Medicine, Monash University, The Alfred Centre, Melbourne, Victoria 3004 Australia; 2 Pragmatic Clinical Trials Unit, Queen Mary University of London, 58 Turner St, London, E1 2AB UK; 3 Medical Statistics Department, London School of Hygiene and Tropical Medicine, Keppel Street, London, WC1E 7HT UK

**Keywords:** Cluster randomised crossover trial, Crossover, Cluster, Reporting quality

## Abstract

**Background:**

The cluster randomised crossover (CRXO) design is gaining popularity in trial settings where individual randomisation or parallel group cluster randomisation is not feasible or practical. Our aim is to stimulate discussion on the content of a reporting guideline for CRXO trials and to assess the reporting quality of published CRXO trials.

**Methods:**

We undertook a systematic review of CRXO trials. Searches of MEDLINE, EMBASE, and CINAHL Plus as well as citation searches of CRXO methodological articles were conducted to December 2014. Reporting quality was assessed against both modified items from 2010 CONSORT and 2012 cluster trials extension and other proposed quality measures.

**Results:**

Of the 3425 records identified through database searching, 83 trials met the inclusion criteria. Trials were infrequently identified as “cluster randomis(z)ed crossover” in title (*n* = 7, 8%) or abstract (*n* = 21, 25%), and a rationale for the design was infrequently provided (*n* = 20, 24%). Design parameters such as the number of clusters and number of periods were well reported. Discussion of carryover took place in only 17 trials (20%). Sample size methods were only reported in 58% (*n* = 48) of trials. A range of approaches were used to report baseline characteristics. The analysis method was not adequately reported in 23% (*n* = 19) of trials. The observed within-cluster within-period intracluster correlation and within-cluster between-period intracluster correlation for the primary outcome data were not reported in any trial. The potential for selection, performance, and detection bias could be evaluated in 30%, 81%, and 70% of trials, respectively.

**Conclusions:**

There is a clear need to improve the quality of reporting in CRXO trials. Given the unique features of a CRXO trial, it is important to develop a CONSORT extension. Consensus amongst trialists on the content of such a guideline is essential.

**Electronic supplementary material:**

The online version of this article (doi:10.1186/s13063-016-1685-6) contains supplementary material, which is available to authorized users.

## Background

The cluster randomised crossover (CRXO) trial design has been used to evaluate a range of interventions, in a variety of settings [1]. In a CRXO trial hospital, schools or other groups of individuals (“clusters”) are randomly assigned to a sequence of interventions. Each cluster receives each intervention at least once in a separate period of time, leading to the formation of “cluster-periods” [2, 3]. The design has potentially greater efficiency than a parallel group cluster randomised trial because the interventions are compared within each cluster [4].

Every trial design has specific features that need to be considered in the design, analysis, and reporting stages. In the case of the CRXO trial, a critical consideration is the correlation between participants within clusters through time. Individuals within a cluster tend to have more similar outcomes than individuals in different clusters. This similarity is typically measured by the within-cluster within-period intracluster correlation (ICC). Furthermore, the similarity between two individuals within a cluster is likely to dissipate as time increases between the measurement of the two individuals. The similarity between two individuals within a cluster, but in different time periods, is typically measured by the within-cluster between-period ICC [2–6].

Complete, transparent, and clear reporting of clinical trials is essential for those using trial reports. Comprehensive reporting allows for an assessment of threats to the validity of the trial results, an assessment of the adequacy of the statistical methods, replication of trial methodologies, incorporation of the trial’s results in synthesis products such as meta-analyses, and implementation of the evaluated intervention(s). To assess the validity of the trial’s results, the methodology should be reported in enough detail to allow for the evaluation of selection, performance, and detection biases [7].

The quality of reporting in randomised trials remains unacceptably low despite the introduction of reporting guidelines [8, 9]. The CONSORT (Consolidated Standards of Reporting Trials) statement reporting guideline for parallel group randomised trials was developed in an attempt to improve the quality of reporting of randomised trials. The CONSORT statement was first published in 1996, and has since been twice revised, first in 2001 [10] and then in 2010 [11]. The 2010 CONSORT statement includes 25 recommended items covering design, conduct, analysis, and other aspects. Extensions to the parallel group CONSORT statement have been published for some alternative designs; however, no extension currently exists for CRXO trials.

While a CONSORT extension is not available for CRXO trials, items from the 2012 cluster trials extension [12] and several items that have been proposed for reporting stepped wedge trials [13] are directly applicable (e.g. ”Allowance for clustering” and ”Allowance for the number of steps” in the sample size justification) or are easily modifiable (e.g. ”Identification as a cluster randomised trial in title”) for CRXO trials. However, the CRXO design has distinct characteristics when compared with the parallel group cluster randomised design and the stepped - wedge design, such as the adverse potential for carryover of the intervention effect to subsequent periods. Therefore, a separate reporting guideline for this trial design may be of value.

Assessing the quality of reporting is the suggested initial step in developing reporting guidelines [9]. Because no published reporting guidelines exist for CRXO trials, in this article we propose possible reporting items, and indicate areas where items may need to be developed, as a means to (1) facilitate discussion on possible items that could be considered for inclusion in a future reporting guideline, and (2) assess the quality of reporting in CRXO trials and thus determine if there is a need for a separate guideline.

To assess the quality of reporting in CRXO trials, we undertook a systematic review that collected information on a range of aspects including trialists’ motivations for using the CRXO design, the design characteristics of CRXO trials, the statistical methods for sample size and data, and the quality of reporting of CRXO design aspects. In a previous publication, we evaluated the appropriateness of the statistical analysis and sample size methods [1]. In this article we evaluate the quality of reporting in CRXO trials.

In “Proposed reporting items for CRXO trials” we discuss potential modifications to the reporting items of the CONSORT 2012 cluster trials extension for CRXO trials, and propose areas where items may need to be developed. In “Systematic review methods” we outline the systematic review methods. The quality of reporting of CRXO trials is presented in the “Results” section. We discuss our findings and conclusions in the “Discussion” section.

## Proposed reporting items for CRXO trials

In this section we suggest, and provide rationale for, possible modifications to reporting items of the CONSORT 2012 cluster trials extension for CRXO trials, and propose areas where items may need to be developed to address the unique design and analysis characteristics of CRXO trials. All CONSORT 2012 cluster trials extension items, proposed modifications, and other indicators of reporting quality are shown in Table 1.

**Table 1:** Quality of reporting of cluster randomised crossover trials as assessed against items from a modified 2012 CONSORT statement extension for cluster randomised trials and selected items from the 2010 CONSORT statement

Section	CONSORT Item no.	CONSORT 2012 extension for cluster trial design for Item no.	Reporting quality assessment measure	Reported? (*N* = 83)
Title and Abstract				
Identification of design in title	1a	Identification as a cluster randomised trial in the title	Identification as a CRXO trial in the title	7 (8%)
Reporting in abstract	1b	See Table 2 [14]	Identification as a CRXO trial in the abstract	21 (25%)
Background and objectives				
Rationale for design	2a	Rationale for using a cluster design	Rationale for using a cluster design AND a crossover of interventions at the cluster level	20 (24%)
Hypothesis and objectives	2b	Whether objectives pertain to the cluster level, the individual participant level or both	No modification proposed	Not assessed
Trial design				
Description of trial design	3a	Definition of cluster and description of how the design features apply to the clusters	Schematic representation of design (recommended especially for designs with >2 periods or interventions)	23 (28%)
			Definition of the cluster	77 (93%)
			Clear differentiation between cluster-period and cluster.	Not assessed
			Number of clusters	79 (95%)
			Number of periods	76 (92%)
			Duration of each time period or when the cross over will occur	Not assessed
			Cohort, repeated cross-sectional, or mixture of designs participants in each period	83 (100%)
			Discussion of the potential for carryover to occur	17 (20%)
			Reporting of use of washout period	83 (100%)
Participants				
Eligibility criteria	4a	Eligibility criteria for clusters	No modification proposed	Not assessed
Interventions				
Description of interventions	5	Whether interventions pertain to the cluster level, the individual participant level or both	No modification proposed	Not assessed
Outcomes				
Description of outcome measures	6a	Whether outcome measures pertain to the cluster level, the individual participant level or both	No modification proposed	Not assessed
Sample size	7a	Method of calculation, number of clusters(s) (and whether equal or unequal cluster sizes are assumed), cluster size, a coefficient of intracluster correlation (ICC or *k*), and an indication of its uncertainty	Was the method for sample size calculation reported, or justification for no sample size calculation provided?	48 (58%)
			Reference to the method used for the sample size calculation	Not assessed
			Justification for number of clusters	33 (40%)
			Justification for number of periods	9 (11%)
			Equal or unequal number of periods per cluster	Not assessed
			Equal or unequal cluster-period sizes	42 (51%)
			A value for the within-cluster within-period ICC or variance components or other measure of correlations within data or justification for not including	13 (16%)
			A value for the within-cluster between-period ICC or variance components or other measure of correlations within data or justification for not including	4 (5%)
			A reference or explanation for the choice of ICCs or other measure of correlations	5 (6%)
			Reported whether the sample size methodology accounted for repeated measurements on the same individual	Not assessed
Sequence generation				
Method used to generate allocation sequence	8a	Method used to generate the random allocation sequence	No modification proposed	36 (43%)
Type of randomisation	8b	Details of stratification or matching if used	Does the article report whether stratified randomisation used?	83 (100%)
Allocation concealment mechanism				
Method used to implement the allocation sequence	9	Specification that allocation was based on clusters rather than individuals and whether allocation concealment (if any) was at the cluster level, the individual participant level, or both	Does the article report whether the people allocating the intervention sequence to the clusters know the allocation sequence?	40 (48%)
			Does the article report whether people recruiting/identifying participants knew which intervention sequence has been assigned to the cluster? (*n* = 57)^a^	44 (77%)
			Does the article report whether the people recruiting/identifying participants could have influenced which people were recruited/identified for inclusion in the study? (*n* = 57)^a^	54 (95%)
Implementation				
Method used to include clusters in trial	10a	Who generated the random allocation sequence, who enrolled clusters, and who assigned clusters to interventions	No modification proposed	Not assessed
Method used to include individuals in clusters	10b	Mechanism by which individual participants were included in clusters for the purposes of the trial (such as complete enumeration, random sampling)	No modification proposed	Not assessed
Method of obtaining consent	10c	From whom consent was sought (representatives of the cluster, or individual cluster members, or both), and whether consent was sought before or after randomisation	From whom was consent sought?	60 (72%)
			Was consent sought before or after randomisation of the cluster when consent was sought from individuals? (*n* = 30)	16 (53%)
Blinding	11a	If done, who was blinded after assignment to interventions (for example, participants, care providers, those assessing outcomes) and how.	Were the participants aware of the intervention assigned to the cluster?	67 (81%)
			Were the researchers who delivered the intervention, i.e. caregiver, aware of the intervention assigned to the cluster?	82 (99%)
			If the outcome was self-reported (*n* = 14), was the participant aware of the intervention assigned to the cluster?	13 (93%)
			If the outcome was assessed by another person (*n* = 69), was the outcome assessor aware of the intervention assigned to the cluster?	45 (65%)
Statistical methods	12a	How clustering was taken into account	Justification for statistical analysis methods	Not assessed
			Reported whether the analysis was performed at the cluster or individual level.	78 (94%)
			Where there are more than two periods, reported whether a single correlation is assumed for the within-cluster between-period correlation	0 (0%)
			Was it possible to determine the method for accounting for both the cluster randomisation and multiple period aspects?	64 (77%)
			Was it possible to determine the method for accounting for the cluster randomisation aspect?	70 (84%)
			Was it possible to determine the method for accounting for the multiple period design aspect?	70 (84%)
Results				
Participant flow				
Number of clusters and participants	13a	For each group, the numbers of clusters that were randomly assigned, received intended treatment, and were analysed for the primary outcome	For each group, reported the number of clusters that were randomly assigned, received intended treatment in each period, and were analysed for the primary outcome	Not assessed
			For each group, reported the number of individuals that were randomly assigned, received the intended intervention in each period, and were analysed for the primary outcome	Not assessed
Losses and exclusions	13b	For each group, losses and exclusions for both clusters and individual cluster members	For each group, losses and exclusions for clusters, cluster-periods, and individual participants	Not assessed
Baseline data	15	Baseline characteristics for the individual and cluster levels as applicable for each group	Presentation of baseline characteristics data in table	
			No baseline characteristics table in article	24 (29%)
			Reported by total only	8 (10%)
			Reported by randomisation sequence with or without total	7 (8%)
			Reported by cluster only	2 (2%)
			Reported by intervention with or without total	37 (45%)
			Reported by cluster and period	2 (2%)
			Reported by intervention and period	1 (1%)
			Reported by intervention, period, and cluster	2 (2%)
Number analysed	16	For each group, number of clusters included in each analysis	For each group, number of clusters, cluster-periods, and participants included in each analysis, stating reasons for exclusions	Not assessed
Outcomes and estimation	17a	Results at the individual or cluster level as applicable and a \coefficient of intracluster correlation (ICC or *k*) for each primary outcome	A coefficient for the within-cluster within-period correlation and within-cluster between-period correlation, or other measure (such as variance components), for each primary outcome	0 (0%)
Generalisability	21	Generalisability to clusters and/or individual participants (as relevant)	No modification proposed	Not assessed

^a^ *n* = 26, no recruitment took place

### Title and abstract (Items 1a, b)

The primary reasons for including a description of the trial design in the title and abstract are to ensure appropriate indexing in electronic databases [12, 14] and to alert the readers to the design so that they are less likely to misinterpret the trial results [14]. A proposed modification is therefore to identify a trial as a “cluster randomised crossover trial” in title and abstract.

### Background and objectives (Item 2a)

Providing a rationale for the trial design in the background informs the reader why the chosen design is best suited to address the research question. The cluster randomisation aspect of the CRXO design typically increases the required number of participants when compared to an individually randomised trial, potentially exposing more participants to harm than necessary if an individually randomised design was feasible [12]. In addition, both the crossover and cluster randomisation aspects of the CRXO design pose trial design, analysis, and implementation challenges. Hence, the choice to use the CRXO design in place of a simpler alternative such as a parallel group cluster randomised trial or individually randomised trial requires justification. Therefore, for a similar reason as proposed in the CONSORT 2012 cluster trials extension [12], we propose that the rationale for the use of cluster randomisation and for the crossover of interventions at the cluster level is included in the background.

### Trial design (Item 3a)

Reporting the trial design allows the reader to replicate the design in future trials and assess whether the implemented sample size and analysis methods were appropriate for the design. We suggest that the following items might be considered important for clearly describing the design of a CRXO trial. Several of these items have been adapted from recommendations for reporting stepped-wedge trials [13]:

- Report the total number of randomised clusters in the trial.
- Report the total number of planned time periods for each cluster in the trial.
- Report the duration of each time period, for example, the duration of time or number of participants included in each cluster-period before the intervention is crossed over.
- Report whether the same, different, or a mix of same and different participants were included in each cluster-period. These designs are described as cohort, repeated cross-sectional, or mixture designs, respectively.
- For complex designs (i.e. designs with more than two interventions and two periods), consider including a schematic representation of the trial design depicting which interventions were allocated to each cluster in each period. For a simple design, the participant flow diagram (Item 13) may suffice.

We propose two new items for CRXO trials:

- Report the potential of the effect of the intervention given in one cluster-period to carry over to subsequent cluster-periods.
- Report methods for managing the risk of carryover, if necessary.

In addition to the above reporting items, we also suggest that articles clearly distinguish between the cluster and the cluster-period.

### Sample size (Item 7a)

Reporting how a sample size calculation has been performed is important for replicability, transparency [12], and scientific and ethical reasons [15]. Reporting of sample size elements of cluster randomised trials has been shown to be incomplete [15]. The sample size calculation for CRXO trials should account for the predicted correlations arising from the design [5, 6]. In addition, the assumed sample size parameters and methodology should be reported. For CRXO trials, we suggest that the following sample size items might be considered important:

- Provide a reference for the sample size methodology or a description of the method when the method is not published.
- Report how the sample size methodology accounts for both the cluster randomisation (e.g. the within-cluster within-period ICC) and the multiple period aspects of the design (e.g. the within-cluster between-period ICC).
- Report how the sample size methodology accounts for whether the same, different, or a mix of the same and different participants will be included in each cluster-period.
- Report the number of clusters, number of periods, and number of participants per cluster-period, noting which are assumed and which are determined by the sample size calculation.
- Report whether a variable or constant number of periods per cluster and participants per cluster-period is assumed.
- Report the parameter values used to account for cluster randomisation and multiple periods.
- Provide a justification for the choice of parameter values and state any constraints on the number of clusters, number of periods, or number of participants per cluster-period.

### Statistical methods (Item 12a)

The primary reasons for reporting the statistical methods are to allow for replication and for the reader to evaluate whether the methods are appropriate for the design [11]. For CRXO trials we suggest that the following items might be considered:

- Provide a reference for the statistical methodology or a description of the method when the method is not published.
- Report whether the analysis was performed at the individual or cluster level.
- Report how both the cluster randomisation and the multiple period aspects of the design were accounted for.
- When there were more than two periods, report whether a constant within-cluster between-period ICC was assumed, and, if a constant within-cluster between-period ICC is not assumed, report what assumption or methodology was used.
- Describe how missing data will be managed at both the individual level and the cluster level [16].

### Participant flow (Items 13a, b)

**Fig. 1:**
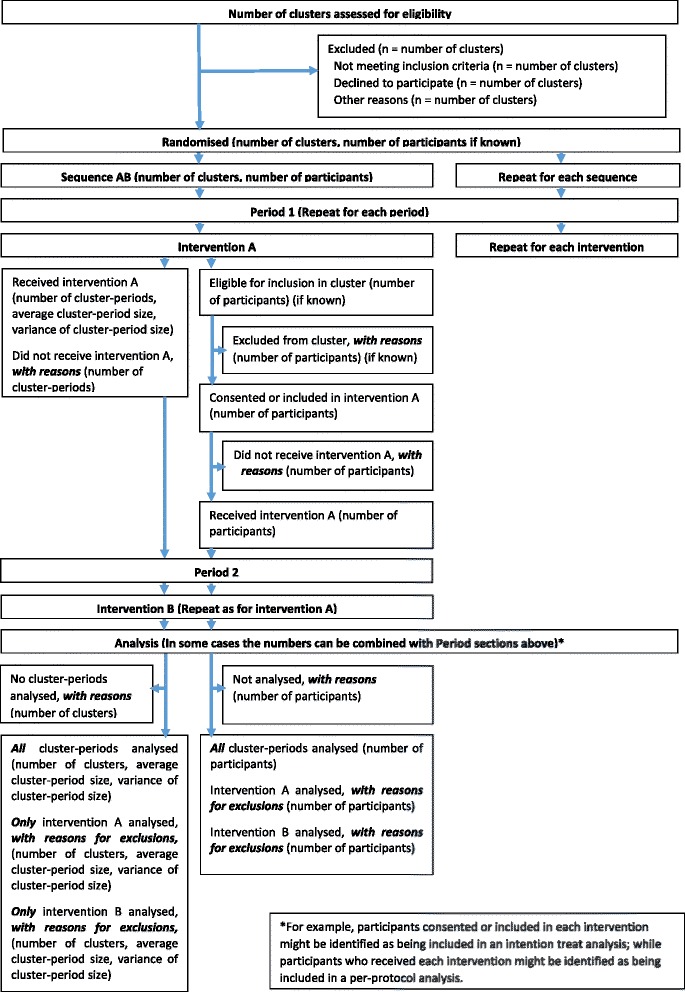
Possible modifications to the CONSORT 2012 cluster trials extension flow diagram (Item 13) for reporting cluster randomised crossover trials

The CONSORT 2012 cluster trials extension [12] notes the importance of providing information on the flow of clusters through the trial (enrolment, allocation, follow-up, analysis) in addition to the flow of participants. A CRXO trial has the added complexity of cluster-periods nested within each cluster and potentially repeated measurements on some participants within each cluster. An additional consideration for reporting is the level at which the analysis is undertaken. The CONSORT 2012 cluster trials extension [12] recommends that if the analysis is aggregated at the cluster level, it is appropriate to show only the flow of clusters through the trial, while for analyses that do not use aggregated data, the flow of individual-level data should also be presented. However, we consider that it is important to show the flow of participants even when the analysis is aggregated at the cluster level, since aggregate-level analyses depend on the individual-level data. To facilitate discussion on presenting the flow of the number of clusters, cluster-period, and participants through a CRXO, we outline possible modifications to the flow diagram in the CONSORT 2012 cluster trials extension [12] and present a possible flow diagram in Fig. 1, although the exact form of the diagram is likely to depend on the trial.

### Baseline data (Item 15)

The main reasons for reporting baseline characteristics are to describe the characteristics of the included population and permit an assessment of the success of the randomisation process. There is additional complexity in a CRXO design, because participants may be recruited to clusters over multiple time periods. Two key considerations when reporting participant and cluster characteristics in a CRXO trial are that (1) randomisation only ensures that, on average, cluster-level characteristics are balanced at baseline (assuming adequate sequence generation and allocation concealment), while individual-level characteristics may be influenced by selection processes; and (2) participants are often recruited at multiple time points, allowing presentation of cluster and individual characteristics at a single time point or summarised across multiple time points.

The cluster-level and individual-level characteristics can be either time invariant or time varying. For example, in a CRXO trial where hospital wards are randomised, the ward type (e.g. surgical, general medical) will remain constant for the duration of the trial. However, other cluster-level characteristics may vary, such as the type of clinicians working on the ward, due to staff changes (e.g. medical students and registrars moving in and out the ward). At the individual level, the characteristics of the individuals are likely to be time varying when new individuals are recruited across the periods (repeated cross-sectional design). However, if the trial is a cohort design, then individual-level characteristics such as sex will remain time invariant, while others, such as weight, may change over the duration of the trial.

We propose that the baseline characteristics are tabulated for each sequence and for each intervention within each sequence. A possible table for a two-period two-intervention CRXO trial is shown in Table 2. This table allows for a number of comparisons to be made for both time-invariant and time-varying characteristics. To facilitate discussion on presenting baseline characteristics in a CRXO trial, we outline a number potential comparisons, many of which have been used in published trials (Table 1), and exemplify these comparisons through Table 2.

**Table 2:** Possible presentation of baseline characteristics in two-period two-intervention cluster randomised crossover trial

Characteristic	Intervention sequence AB		Intervention sequence BA	
	Period 1–Intervention A Group 1	Period 2–Intervention B Group 2	Period 1–Intervention B Group 3	Period 2–Intervention A Group 4
Time-invariant characteristics				
Time-invariant cluster characteristic	Such as proportion of each ward type: Cardiac: 25% Intensive Care: 40% Neurology: 35%	Such as proportion of each ward type: Cardiac: 25% Intensive Care: 40% Neurology: 35%	Such as proportion of each ward type: Cardiac: 35% Intensive Care: 45% Neurology: 20%	Such as proportion of each ward type: Cardiac: 35% Intensive Care: 45% Neurology: 20%
Time-invariant participant characteristic (cohort design only)	Such as patient sex: 59% male	Such as patient sex: 59% male	Such as patient sex: 48% male	Such as patient sex: 48% male
Time-varying characteristics				
Time-varying cluster characteristic	Nurse-to-patient ratio over 24 h, Median (IQR): 2.1 (2.0 – 2.2)	Nurse-to-patient ratio over 24 h, Median (IQR): 2.0 (1.9 – 2.1)	Nurse-to-patient ratio over 24 h, Median (IQR): 2.3 (2.1 – 2.4)	Nurse-to-patient ratio over 24 h, Median (IQR): 2.2 (2.1 – 2.4)
Time-varying participant characteristic (cohort and repeated cross-sectional design)	Such as patient weight (kg), Mean (SD): 83.4 (14.2)	Such as patient weight (kg), Mean (SD): 78.9 (15.6)	Such as patient weight (kg), Mean (SD): 81.2 (13.2)	Such as patient weight (kg), Mean (SD): 80.4 (11.2)

#### Time-invariant characteristics

The time-invariant characteristics are described as follows:

1. Compare time-invariant characteristics of clusters allocated to sequence AB (Group 1 + Group 2) with clusters allocated to sequence BA (Group 3 + Group 4).
2. Compare time-invariant characteristics of participants recruited to sequence AB (Group 1 + Group 2) with participants recruited to sequence BA (Group 3 + Group 4) (cohort design).

Comparison 1 allows the success of the randomisation process to be evaluated. Comparison 2 allows for the process of recruiting participants into clusters to be evaluated. When the number of clusters is small, then chance imbalances between sequences may occur.

3. Compare time-invariant characteristics of clusters in all periods allocated to intervention A (Group 1 + Group 4) with clusters allocated to intervention B (Group 2 + Group 3).
4. Compare time-invariant characteristics of participants recruited to intervention A (Group 1 + Group 4) with participants recruited to intervention B (Group 2 + Group 3) (cohort design).

Comparisons 3 and 4 are equivalent to comparisons 1 and 2 when there is no loss of clusters or participants over time.

#### Time-varying characteristics

The time-varying characteristics are described as follows:

5. Compare time-varying characteristics of clusters allocated to sequence AB (Group 1 + Group 2) with clusters allocated to sequence BA (Group 3 + Group 4).
6. Compare time-varying characteristics of participants recruited to sequence AB (Group 1 + Group 2) with participants recruited to sequence BA (Group 3 + Group 4) (cohort and repeated cross-sectional design).

Comparisons 5 and 6 are likely to be of limited value. Presenting cluster-level characteristics summarised over multiple time periods can obscure whether randomisation was successful if systematic changes have occurred within the clusters. Likewise, presenting individual-level characteristics summarised over multiple time periods can obscure whether systematic changes have occurred in the recruitment of participants within the clusters.

7. Compare time-varying characteristics of clusters in all periods allocated to intervention A (Group 1 + Group 4) with clusters allocated to intervention B (Group 2 + Group 3).
8. Compare time-varying characteristics of participants recruited to intervention A (Group 1 + Group 4) with participants recruited to intervention B (Group 2 + Group 3) (cohort and repeated cross-sectional design).

As for comparisons 5 and 6, comparisons 7 and 8 also summarise cluster-level and individual-level characteristics over multiple time periods.

9. Compare time-varying characteristics of clusters allocated to intervention A with clusters allocated to intervention B, in the first period only (Group 1 vs Group 3).
10. Compare time-varying characteristics of participants recruited to intervention A with participants recruited to intervention B, in the first period only (Group 1 vs Group 3) (cohort and repeated cross-sectional design).

The considerations for comparisons 1 and 2 apply also to comparisons 9 and 10. However, comparisons 9 and 10 do not allow any evaluation of change in the characteristics over time and do not consider all participant data.

11. Compare characteristics of clusters allocated to intervention A with clusters allocated to intervention B, separately for each sequence (Group 1 vs Group 2 AND Group 3 vs Group 4).
12. Compare characteristics of participants recruited to intervention A with participants recruited to intervention B, separately for each sequence (Group 1 vs Group 2 AND Group 3 vs Group 4) (cohort and repeated cross-sectional design).
13. Compare characteristics of clusters allocated to intervention A with clusters allocated to intervention B, separately for each period (Group 1 vs Group 3 AND Group 2 vs Group 4).
14. Compare characteristics of participants recruited to intervention A with participants recruited to intervention B, separately for each period (Group 1 vs Group 3 AND Group 2 vs Group 4) (ohort and repeated cross-sectional design).

Presentation of cluster-level and individual-level characteristics separately by period in each intervention (comparisons 11–14) allows for assessment for any systematic change in characteristics over time or any potential interaction between intervention and time. Such changes will be obscured by presenting characteristics summarised over multiple time periods.

### Number analysed (Item 16)

Reporting the number of clusters, cluster-periods, and participants that contribute to each analysis of each outcome is essential to interpreting the results. To facilitate discussion on presenting the numbers analysed in CRXO trials, we outline a potential approach:

- Present the number of clusters, cluster-periods, and participants analysed for the primary outcome as per the participant flow diagram (Fig. 1).

In addition, for each secondary analysis and outcome, either state that the same clusters, cluster-periods, and individuals are included as in the primary analysis, or where the number analysed differs from Fig. 1:

- Report the number of clusters that contribute to the analysis across all periods, also separately by intervention sequence, and give reasons for the exclusion of any whole clusters.
- Report the number of clusters that contribute to the analysis for only some periods, also separately by intervention and intervention sequence. Give reasons for the exclusion of any cluster-periods and state whether the remaining clusters-periods from that cluster were included.
- Report the number of participants included in the analysis, by intervention and intervention sequence, including the reasons for any exclusions at the individual level.

### Outcomes and estimation (Item 17a)

The importance of providing estimates of within-cluster correlation in cluster randomised trials for the purpose of describing the clustering and future sample size estimation is well recognised [12]. For similar reasons, in CRXO trials it is important to provide estimates of within-cluster between-period ICCs, in addition to the within-cluster within-period ICCs for each outcome. Alternatively, if mixed models are used, the reporting of variance components can be provided from which the ICCs can be calculated.

## Methods

The protocol for the review has been published [17]. Here we provide only a brief overview of the methods, along with deviations from the planned methods, and outline the measures used to assess reporting quality.

### Literature search

In brief, MEDLINE, PubMed, EMBASE, and CINAHL Plus were searched until December 2014 for English language articles of CRXO trials. In addition to searching for CRXO trials, we searched PubMed for CRXO methodology articles to identify further references to CRXO trials. A citation search of all identified methodology articles was performed in Web of Science. Finally, the references of all eligible articles were screened for CRXO trials. No restriction was applied to the publication date. The search strategies for CRXO trials and CRXO methodology articles are outlined in Arnup et al. [17] and provided in Additional file 1.

### Trial inclusion criteria

Trials that met the following inclusion criteria were included in the review: the trial was undertaken in humans; the allocation of the intervention was to clusters of individuals rather than individuals themselves; each cluster received each intervention in a sequence over time (conventional crossover design), or at least some clusters crossed over from one intervention to another (such as two-treatment-four-sequence designs AA, AB, BA, and BB); at least some clusters crossed each way between at least two interventions (e.g. one cluster received AB and one cluster received BA and therefore excludes pre-post designs); and the intervention given in the one period was not deliberately intended by design to affect individuals in subsequent periods (e.g. interventions intended to change the prescribing behaviour of health care providers). The latter two criteria were added while undertaking the review. Protocols were included in the review; however, for this article the focus is on the quality of reporting of trial reports, and hence protocols have been excluded.

### Selection of trials for inclusion in the review

One author (SA) assessed all titles and abstracts using the eligibility criteria, and 50% of the titles and abstracts were screened independently by at least one co-author. All full-text articles were then assessed by one author (SA) using the eligibility criteria. Of these, all eligible articles were double screened, along with 20% of articles that were initially determined to be ineligible. Differences in inclusion decisions were resolved by discussion or by referral to a third author. No ineligible articles were subsequently found to be eligible.

### Data extraction and management

One author (SA) extracted data from all trials, and data from 20% of the trials were independently double data extracted by the co-authors. Three of the five authors (SA, JM, AF) reviewed the discrepancies arising from the double data extraction and discussed processes for further reviewing items where there was inconsistency. The processes and items where further review was undertaken are described in Arnup et. al. [17]. The data extraction form was piloted on five trials by each author. Data were entered into a database (Microsoft Access 2010, Redmond, Washington, USA).

To examine the reporting quality of the CRXO trials, we extracted reported information from the trials on selected 2012 cluster trial CONSORT extension items [12], with modification so that they were suitable to assess CRXO trials (Items 1a,b and Item 2a of the 2012 cluster trials extension [12]; hereafter we only refer to the item number). Where the CONSORT extension may not have adequately covered the unique characteristics of CRXO trials, we extracted information on indicators of the reporting quality for that item (Item 3a, Item 7a, Item 8b, Item 9, Item 10c, Item 12a, Item 15, Item 17a). We refer to these measures as indicators because further discussion between trialists using the CRXO design is required to determine if the measure adequately assesses reporting quality.

We did not extract information on CONSORT items where the reporting considerations did not differ from a parallel group cluster trial or individually randomised trial, e.g. description of the interventions and outcome measures (Item 2b, Item 3b, Items 4a, b, Item 5, Items 6a, b, Item 7b, Items 10a, b, Item 11b, Item 12b, Items 14a, b, Item 17b, Items 18–25.). However, there were two exceptions where we did extract information on the following 2010 CONSORT items [11]: Item 8a “Method used to generate random allocation sequence” because the item is required to evaluate the potential for selection bias, and Item 11a “Who was blinded after assignment to intervention?” because the item was required to evaluate the risk of detection bias (see the next paragraph). For Item 13 “Participant flow” and Item 16 “Number analysed”, we present a discussion of possible reporting approaches only.

In addition to assessing the quality of reporting of CRXO trials against reporting items and indicators, we assessed whether the reported information was sufficient to judge the risk of selection, performance, and detection bias. The *Cochrane Handbook for Systematic Reviews of Interventions*, Section 8.4, defines selection bias as systematic differences between baseline characteristics of the groups that are compared; performance bias as systematic differences between groups in the care that is provided, or in exposure to factors other than the interventions of interest; and detection bias as systematic differences between groups in how outcomes are determined. (See Higgins JPT, Green S (editors). *Cochrane Handbook for Systematic Reviews of Interventions* Version 5.1.0 [updated March 2011]. The Cochrane Collaboration, 2011. Available from http://handbook.cochrane.org/.)

The full list of extracted data is available in Arnup et al. [17]. The extracted data specific to reporting quality in CRXO trials were: identification of the design in the title or abstract; justification for using the design; selected design characteristics including schematic representation of the design, definition of the cluster, number of clusters, number of periods, type of design (cohort, repeated cross-sectional, or mixture of designs), and management of the risk of carryover of intervention effects between periods; reporting of sample size calculation details including justification for the number of clusters, justification for the number of periods, equal or unequal cluster-period sizes, the assumed measure of similarity between the outcomes of individuals within a cluster within a given period and justification for assumption, and the reported measure of similarity between outcomes of individuals within a cluster between different periods and justification for assumption; methods used in the trial including recruitment, consent, randomisation, allocation, and blinding; statistical analysis including level of analysis, the method accounting for the similarity between the outcomes of individuals within a cluster within a given period and the similarity between outcomes of individuals within a cluster between different periods, and the reported measure of similarity between outcomes of individuals within a cluster between different periods; losses and exclusions of participants; and reporting of baseline characteristics.

### Data coding

In the section, we provide details on how we judged each reporting quality measure.

We classified the following items as reported if a clear statement addressing the item was provided in the trial report: Items 1a, b, Item 2a, Item 10c, Item 12a, Item 15, and Item 17a.

For items that were not explicitly reported in the trial report, we reviewed the reported methods to determine whether enough information was provided to classify the following items as reported: same, different, or a mix of participants in each period (Item 3a); equal or unequal cluster sizes in the sample size calculation (Item 7a); use of restricted randomisation (Item 8b); items addressing allocation concealment (Item 9); and blinding (Item 11) methods.

We classified the method of random allocation (Item 8a) as “reported” if the article included details on how the random allocation was achieved or clearly stated that the allocation was not random. The method of random allocation in articles that stated that the allocation was “at random”, with no further detail, was classified as “not reported”.

We classified the information reported in each trial as either sufficient or insufficient to assess the risk of selection bias, performance bias, and detection bias. To assess whether sufficient information was reported to judge the risk of selection bias, we required that enough detail was reported to assess (1) whether the researcher allocating the cluster to the intervention sequence was blind to future allocation assignments (Item 9); (2) whether the people recruiting or identifying participants knew which intervention sequence had been assigned to the cluster (Item 9); (3) whether the researcher recruiting or identifying participants could influence which individuals were included in the trial (Item 9); and if the individual participant (or other person on their behalf) provided consent, whether they had knowledge, prior to consenting, of the intervention assigned to the cluster (Item 10c). To assess whether sufficient information was reported to judge the risk of performance bias, we required that enough detail was reported to assess if either the participants or those delivering the intervention (e.g. health care professionals) were aware of the intervention (Item 11a). To assess whether sufficient information was reported to judge the risk of detection bias, we required that enough detail was reported to determine if the assessment of outcomes was self-reported or measured by another person and whether the assessor was aware of the intervention assigned to the cluster (Item 11a).

### Data analysis

We present descriptive summary statistics using frequencies and percentages of responses to categorical data. Free text was classified and frequencies and percentages of the categories are presented. The extracted data from individual trials can be made available upon request to the corresponding author.

## Results

### Results of the search

**Fig. 2:**
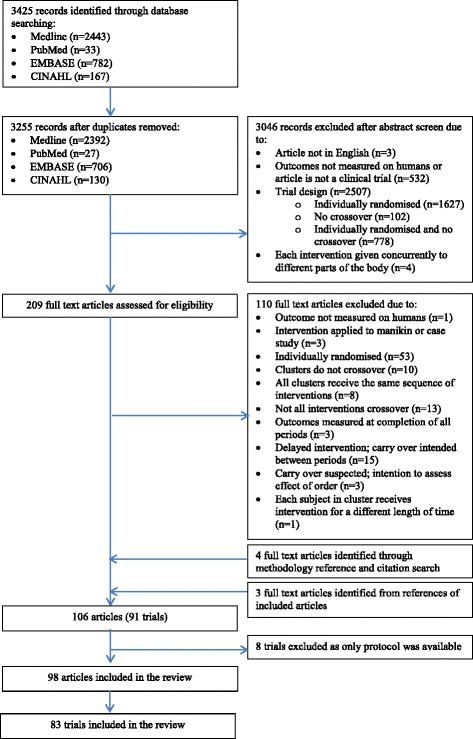
Flow of articles through the systematic review

Figure 2 shows the flow diagram of the CRXO trial selection process for the systematic review. Of the 3425 records identified through database searching, 170 were duplicates and 3046 were ineligible based on screening of abstracts, leaving 209 full-text articles to assess for eligibility. Of these 209 articles, 99 were assessed as eligible. A further four articles were identified through the methodology article reference and citation search, and three articles from the references of eligible articles. In this article we further exclude eight trials where only a protocol was available. In total, 98 articles from 83 trials were included in this paper. Seventy-one trials had only one associated article, nine trials had two associated articles, and three trials had three associated articles.

### Characteristics of the trials

Most trials were conducted in a developed country (*n* = 79, 95%) and were undertaken within a single country (*n* = 80, 96%) (Additional file 2: Table S1). The types of clusters varied, with almost half of the trials (*n* = 40, 48%) randomising hospitals or wards within hospitals, 13 (16%) randomising individual health care providers, and 11 (13%) randomising schools or classes (Additional file 3: Table S2).

The trials investigated a wide range of diseases and conditions and health care delivery models. Nineteen trials (23%) investigated infection control, ten (12%) investigated cardiovascular disease, nine (11%) examined the delivery of health services, and eight (10%) investigated infectious diseases (Table 3).

**Table 3:** Characteristics of the cluster randomised crossover trials

Disease or domain under study	*n* (%) (*N* = 83)
Infection control	19 (23%)
Cardiovascular disease	10 (12%)
Health services delivery	9 (11%)
Infectious disease	8 (10%)
General and public health	5 (6%)
Medical training	5 (6%)
Communication of health information	4 (5%)
Pregnancy, childbirth, and early childhood	3 (4%)
Mental health and behavioural conditions	3 (4%)
Respiratory disease	3 (4%)
Blood sample contamination	3 (4%)
Cognition	3 (4%)
Central nervous system and musculoskeletal disease	2 (2%)
Oral health	2 (2%)
Nutritional and metabolic disorders	1 (1%)
Urogenital disease	1 (1%)
Digestive disorders	1 (1%)
Pain management	1 (1%)
Type of intervention	
Intervention targeting the individual	42 (51%)
Intervention targeting health care provider	21 (25%)
Quality improvement intervention	14 (17%)
Intervention resulting in change to the participant environment	6 (7%)
Number of interventions	
2	74 (89%)
3	8 (10%)
4	1 (1%)
Number of clusters - Median [IQR]; Range	8 [3–21]; 2 – 268
Unclear	4 (5%)
Number of periods^a^	
2	53 (70%)
3	8 (11%)
4+	15 (18%)
Unclear	7 (8%)
Cluster-period size - Median [IQR]; Range	27 [14–77]; 2 – 1319
Unclear	17 (20%)

^a^Percentages of non-missing data presented

The most common trial design involved two interventions (*n* = 74, 89%). The majority of trials (70%) used two periods. Trials had a median of eight clusters (IQR: 3 – 21, range: 2– 268) and a median cluster-period size of 27 (IQR: 14–77, range: 2–1319) (Table 2).

In 42 trials (51%) the interventions were delivered directly to the individuals within the clusters. In 21 trials (25%) the intervention was targeted at the health care provider rather than the individuals under their care, and in 14 trials (17%) the intervention was targeted at the organisation of the health care provider or health services delivery (Table 2).

### Quality of reporting in CRXO trials as assessed against proposed or modified reporting items and other indicators

Trials were infrequently identified as “Cluster Randomised Crossover” trials in the title (8%) or in the abstract (25%). A rationale for both the cluster randomisation and crossover aspects of the design was provided in 20 trials (24%). Most design characteristics were reported; however, trials infrequently used a schematic to illustrate the design (28%), even in designs with either more than two periods or more than two interventions (30%, *n* = 7/23) (Table 1).

The reporting of the methods used to generate the allocation sequence and assign the allocation sequence to clusters was incomplete in 43% (*n* = 36) and 48% (*n* = 40) of trials, respectively. In 20% (*n* = 17) of the trials, the risk of a carryover of the intervention effect from one period to subsequent periods was discussed (Table 1).

Reporting of the methods and parameters to calculate the sample size was often missing or incomplete. Only 48 trials (58%) provided a sample size calculation or justification for not performing a sample size calculation. Thirty-three trials (40%) provided justification for the number of clusters, and only nine trials (11%) provided justification for the number of periods. Of the 83 trials, only 13 (16%) reported the within-cluster within-period ICC, and only four (5%) reported the within-cluster between-period ICC (or corresponding variance components) that was assumed in the sample size calculation (Table 1).

The construction of the baseline characteristics tables of the individual participants varied. In most trials, the characteristics were reported by intervention group (45%); some trials reported by intervention sequence (8%). In 24 trials (29%), no baseline characteristics table was presented (Table 1).

Most trials gave sufficient detail to determine whether the analysis was performed at the level of the individual or the level of the cluster (*n* = 78, 94%). However, in 19 trials (23%) it could not be determined how or whether the analysis accounted for the cluster randomisation or multiple period aspects of the design. No trial reported a measure of both intracluster correlations or variance components induced by the cluster randomisation and multiple period aspects of the design (Table 1). One trial reported the within-cluster ICC from an analysis that included only a random effect for cluster, therefore assuming that the within-cluster between-period ICC was equal to the within-cluster within-period ICC.

### Quality of reporting in CRXO trials to allow assessment of bias

#### Selection bias

Twenty-five trials (30%) provided sufficient information to assess the risk of selection bias (Table 4). In 43 trials (52%) we were unable to judge the risk of selection bias because we could not determine whether the researchers responsible for allocating the intervention sequence to the clusters were aware, or not, of the intervention sequence; in 13 trials (17%) we were unable to judge the risk of selection bias because it was not clear whether the researchers recruiting participants were aware, or not, of the cluster’s intervention sequence; in three trials (4%) we were unable to judge the risk of selection bias because we could not judge whether the researchers responsible for recruiting/identifying participants were able to influence recruitment; and in 14 of the 30 trials (47%) where individual consent was sought, we could not assess the risk of selection bias because we could not judge whether the participant was aware of the intervention assigned to the cluster prior to giving consent.

**Table 4:** Quality of reporting in CRXO trials of material required to assess selection, performance, and detection biases

Bias	Sufficient information to evaluate risk of bias (*N* = 83)
Selection bias	25 (30%)
Performance bias	67 (81%)
Detection bias	58 (70%)

#### Performance bias

Sixty-seven trials (81%) provided sufficient information to assess performance bias (Table 4). In the 16 trials (19%) that did not provide sufficient detail to assess the risk of performance bias, we could not judge whether the intervention was concealed, or not, from the participants. In one trial (1%) we also could not judge whether the intervention was concealed, or not, at cluster level.

#### Detection bias

Fifty-eight trials (70%) provided sufficient information to assess detection bias (Table 4). Of the 14 trials (17%) in which the primary outcome was self-report, we could not judge if the participant was aware, or not, of the intervention assigned to the cluster-period in one trial (7%). Of the 69 trials (83%) where the primary outcome was not self-report, we could not judge if the assessor was aware, or not, of the intervention in 24 trials (35%).

## Discussion

We proposed possible reporting items for CRXO trials as a basis for further discussion and to examine reporting quality. The items were either modified from those in the 2010 CONSORT [11] and 2012 cluster trial extension statements [12] or were proposed reporting indicators. Incomplete reporting of the design aspects that are unique to the CRXO design was found to be common in the published trials included in the systematic review.

The frequency of reporting of sample size calculations was similar in CRXO trials compared with other randomised trial designs, including individually randomised trials, parallel group cluster randomised trials, individual crossover trials, and stepped - wedge trials [8, 15, 18–21]. Reporting of the ICCs assumed in the sample size calculation was poorer in CRXO trials compared with parallel group cluster randomised trials, with the within-cluster within-period ICC and within-cluster between-period ICC assumed in the sample size calculation only reported in 5% of CRXO trials compared with 35% in parallel group cluster randomised trials [15]. Furthermore, no CRXO trial reported both the ICC observed in the analysis and the ICC assumed in the sample size calculation, compared with 11% of parallel group cluster randomised trials [15].

The completeness of reporting risk of bias domains for CRXO trials was better than previously observed estimates for the domains: method of random sequence generation, method of allocation concealment, and blinding [8, 18, 19, 21]. This more complete reporting may reflect our generous assessment of complete reporting for these domains, or the greater number of trials in this review that were published after the publication of the 2010 CONSORT statement [11] and 2012 cluster trials extension [12]. However, these domains were still incompletely reported in around half of CRXO trials.

Complete reporting of individually randomised crossover trials allows identification of the potential for carryover and of the methods used to manage potential carryover, including the use of washout periods. While we were able to judge if a washout period had been used in all CRXO trials, discussion of the potential for carryover only occurred in 20% of trials included in this systematic review. This estimate was similar to that observed in a study examining the reporting of individually randomised crossover trials (29%) [19]. Previous estimates for the reporting of the use of a washout period include 70% [19] and 99% [20].

Assessing the quality of reporting of published CRXO trials is a recommended initial step in developing reporting guidelines [9]. This should be undertaken in combination with reviewing relevant existing guidelines to determine whether it is most appropriate to amend an existing guideline or develop a new guideline. The CRXO design has unique features, and reporting guidance for these features is currently not addressed by items in existing guidelines [11–13]. Therefore, it was necessary to concurrently propose reporting items and assess the quality of reporting against these items.

The results of the present study suggest a need for improved reporting of CRXO trials, and given the lack of specific guidance for this design, a CONSORT extension would be of value. Recommended next steps would include setting up a consensus process, including participants with relevant expertise, to decide upon the specific items and their wording [9]. However, in the absence of specific guidance for this design, our suggested modifications may usefully inform reporting of CRXO trials until formal guidelines are developed.

### Strengths and limitations

Our review represents the most comprehensive review of this trial design to date, despite some potential limitations in the methods used to locate CRXO trials, which have been previously discussed [1]. In brief, it may be argued that better reported trials are easier to locate, and thus, our results may present an optimistic view of the reporting quality in CRXO trials.

Our conclusions of the reporting quality in CRXO trials may also depend on our chosen reporting quality measures. However, our reporting quality items were predefined, and were based on items modified from the 2010 CONSORT statement [11] and 2012 cluster trials extension [12] wherever possible. However, the next step would be to undertake a more rigorous process to refine and agree upon the reporting items using a consensus process such as the Delphi method.

## Conclusions

We have proposed possible reporting items for CRXO trials as a basis for further discussion and to examine reporting quality. We found that incomplete reporting of the design aspects that are unique to the CRXO design was common in the published trials included in this systematic review. Given these results, it is important that a CONSORT extension is developed. Consensus amongst trialists on the content of such a guideline is essential.

## Additional files

Additional file 1: Search strategies. Appendix containing the search strategies to identify CRXO trials. (DOCX 12 kb)
Additional file 2: Table S1. Country where the trial was conducted. Table containing additional demographic data of the included trials in the review. (DOCX 12 kb)
Additional file 3: Table S2. Type of randomised cluster. Table containing additional demographic data of the included trials in the review. (DOCX 12 kb)

## Abbreviations

CONSORT: CONsolidated Standards Of Reporting Trials
CRXO: Cluster randomised crossover
ICC: Intracluster correlation
IQR: Interquartile range
SD: Standard deviation

## References

[1] Arnup SJ, Forbes AB, Kahan BC, Morgan KE, McKenzie JE (2016). Appropriate statistical methods were infrequently used in cluster-randomized crossover trials. J Clin Epidemiol..

[2] Turner RM, White IR, Croudace T (2007). Analysis of cluster randomized cross-over trial data: a comparison of methods. Stat Med.

[3] Parienti JJ, Kuss O (2007). Cluster-crossover design: a method for limiting clusters level effect in community-intervention studies. Contemp Clin Trials.

[4] Rietbergen C, Moerbeek M (2011). The design of cluster randomized crossover trials. J Educ Behav Stat.

[5] Giraudeau B, Ravaud P, Donner A (2008). Sample size calculation for cluster randomized cross-over trials. Stat Med.

[6] Forbes AB, Akram M, Pilcher D, Cooper J, Bellomo R (2015). Cluster randomised crossover trials with binary data and unbalanced cluster sizes: application to studies of near-universal interventions in intensive care. Clin Trials.

[7] Higgins JPT, Green S, editors. Cochrane handbook for systematic reviews of interventions version 5.1.0 [updated March 2011]. Oxford: The Cochrane Collaboration; 2011. Available from http://handbook.cochrane.org/.

[8] Hopewell S, Dutton S, Yu LM, Chan AW, Altman DG (2010). The quality of reports of randomised trials in 2000 and 2006: comparative study of articles indexed in PubMed. BMJ..

[9] Moher D, Schulz KF, Simera I, Altman DG (2010). Guidance for developers of health research reporting guidelines. PLoS Med.

[10] Moher D, Schulz KF, Altman D, Group C (2001). The CONSORT statement: revised recommendations for improving the quality of reports of parallel-group randomized trials. JAMA.

[11] Schulz KF, Altman DG, Moher D, Group C (2010). CONSORT 2010 statement: updated guidelines for reporting parallel group randomised trials. J Clin Epidemiol.

[12] Campbell MK, Piaggio G, Elbourne DR, Altman DG, Group C (2012). Consort 2010 statement: extension to cluster randomised trials. BMJ..

[13] Hemming K, Haines TP, Chilton PJ, Girling AJ, Lilford RJ (2015). The stepped wedge cluster randomised trial: rationale, design, analysis, and reporting. BMJ..

[14] Hopewell S, Clarke M, Moher D, Wager E, Middleton P, Altman DG, Schulz KF, Group C (2008). CONSORT for reporting randomized controlled trials in journal and conference abstracts: explanation and elaboration. PLoS Med.

[15] Rutterford C, Taljaard M, Dixon S, Copas A, Eldridge S (2015). Reporting and methodological quality of sample size calculations in cluster randomized trials could be improved: a review. J Clin Epidemiol.

[16] Giraudeau B, Ravaud P (2009). Preventing bias in cluster randomised trials. PLoS Med.

[17] Arnup SJ, Forbes AB, Kahan BC, Morgan KE, McDonald S, McKenzie JE (2014). The use of the cluster randomized crossover design in clinical trials: protocol for a systematic review. Syst Rev..

[18] Ivers NM, Taljaard M, Dixon S, Bennett C, McRae A, Taleban J, Skea Z, Brehaut JC, Boruch RF, Eccles MP (2011). Impact of CONSORT extension for cluster randomised trials on quality of reporting and study methodology: review of random sample of 300 trials, 2000-8. BMJ..

[19] Mills EJ, Chan AW, Wu P, Vail A, Guyatt GH, Altman DG (2009). Design, analysis, and presentation of crossover trials. Trials..

[20] Li T, Yu T, Hawkins BS, Dickersin K (2015). Design, analysis, and reporting of crossover trials for inclusion in a meta-analysis. PLoS One.

[21] Mdege ND, Man MS, Taylor Nee Brown CA, Torgerson DJ (2011). Systematic review of stepped wedge cluster randomized trials shows that design is particularly used to evaluate interventions during routine implementation. J Clin Epidemiol.

