# Supplementary material for: The quality of reporting in cluster randomised crossover trials: proposal for reporting items and an assessment of reporting quality
Source: Trials. 2016 Dec 6;17:575. doi: 10.1186/s13063-016-1685-6 (PMC5142135; doi:10.1186/s13063-016-1685-6)
Supplement: Additional file 1: — Search strategies. Appendix containing the search strategies to identify CRXO trials. (DOCX 12 kb) [file 13063_2016_1685_MOESM1_ESM.docx]

**Search strategies**

**Ovid MEDLINE search**

CROSS OVER TERMS

1. (cross-over or cross?over or "cross* over").tw.

2. (switch-over or switch?over or "switch* over" or switch-back or switch?back or "switch* back" or switched).tw.

3. ((change-over or change?over or "change* over") not ((change-over or change?over or "change* over") adj1 time)).tw.

4. (ab*ba* adj3 design*).tw.

5. exp Cross-Over Studies/

6. 1 or 2 or 3 or 4 or 5

CLUSTER ALLOCATION TERMS

7. ((unit$1 or school$1 or hospital$1 or cluster* or region$1 or ward* or practice* or communit* or population* or facility or facilities or practitioner*) adj15 random*).tw.

8. ((unit$1 or school$1 or hospital$1 or cluster* or region$1 or ward* or practice* or communit* or population* or facility or facilities or practitioner*) adj15 interven*).tw.

9. ((group* adj random*) or (group* adj interven*)).tw.

10. 7 or 8 or 9

HUMANS ONLY

11. Humans/

12. Animals/

13. 12 not 11

COMBINE CONCEPTS

14. 6 and 10

15. 14 not 13

**PubMed search**

CROSS OVER TERMS

1. "cross-over"[tiab] OR crossover[tiab] OR "cross over" [tiab] OR "crossed over"[tiab]

2. "switch-over"[tiab] OR switchover[tiab] OR "switch over"[tiab] OR "switch-back"[tiab] OR switchback[tiab] OR "switch back"[tiab] OR switched[tiab]

3. (change-over[tiab] OR changeover[tiab] OR "change over"[tiab] OR "changed over"[tiab] OR "changes over"[tiab]) not ("change-over time"[tiab] OR "changeover time"[tiab] OR "change over time"[tiab] OR "changed over time"[tiab] OR "changes over time"[tiab])

4. ab*ba[tiab]

5. Cross-Over Studies[mh]

6. #1 OR #2 OR #3 OR #4 OR #5

CLUSTER ALLOCATION TERMS

7. (cluster-randomi*[tiab] OR “cluster randomised”[tiab] OR “cluster randomized”[tiab] OR “cluster randomization”[tiab] OR “cluster randomisation”[tiab])

HUMANS ONLY

8. (Animals[mh] NOT Humans[mh])

COMBINE CONCEPTS

9. #6 AND #7

10.#9 NOT 8

11.#10 NOT MEDLINE[sb]

**EMBASE search via embase.com**

CROSS OVER TERMS

1. (cross-over or crossover or "cross over" or "crosses over" or "crossed over" or "crossing over"):ti:ab

2. (switch-over or switchover or "switch over" or "switches over" or "switched over" or switch-back or "switchback" or "switch back" or "switches back" or "switched back" or switched):ti:ab

3. ((change-over or changeover or "change over" or "changes over" or "changed over") not ((change-over or changeover or "change over" or "changes over" or "changed over") near/1 time)):ti:ab

4. (abba near/3 design):ti:ab or (abba near/3 designs):ti:ab

5. “crossover procedure”/exp

6. #1 or #2 or #3 or #4 or #5

CLUSTER ALLOCATION TERMS

7. ((unit or units or school or schools or hospital or hospitals or cluster or clusters or region or regions or ward or wards or practice or practices or community or communities or population or populations or facility or facilities or practitioner or practitioners) near/15 (random or randomly or randomise or randomize or randomised or randomized or randomises or randomizes or randomisation or randomization)):ti:ab

8. ((unit or units or school or schools or hospital or hospitals or cluster or clusters or region or regions or ward or wards or practice or practices or community or communities or population or populations or facility or facilities or practitioner or practitioners) near/15 (intervene or intervention or interventions)):ti:ab

9. ((group or groups or grouped) near/1 (random or randomly or randomise or randomize or randomised or randomized or randomises or randomizes or randomisation or randomization)):ti:ab or ((group or groups or grouped) near/1 (intervene or intervention or interventions)):ti:ab

10. #7 or #8 or #9

HUMANS ONLY

11. ‘animal’ not ‘human’

COMBINE CONCEPTS

12. #6 and #10

13. #12 not #11

14. #13 not ‘medline’

**CINAHL Plus search**

CROSS OVER TERMS

1. TI ( ("cross-over" or "cross?over" or "cross* over") ) OR AB ( ("cross-over" or "cross?over" or "cross* over") )

2. TI ( ("switch-over" or "switch?over" or "switch* over" or "switch-back" or "switch?back" or "switch* back" or switched) ) OR AB ( ("switch-over" or "switch?over" or "switch* over" or "switch-back" or "switch?back" or "switch* back" or switched) )

3. TI ( (("change-over" or "change?over" or "change* over") not (("change-over" or "change?over" or "change* over") n1 time)) ) OR AB ( (("change-over" or "change?over" or "change* over") not (("change-over" or "change?over" or "change* over") n1 time)) )

4. TI (ab*ba* n3 design*) OR AB (ab*ba* n3 design*)

5. (MH "Crossover Design")

6. S1 or S2 or S3 or S4 or S5

CLUSTER ALLOCATION TERMS

7. TI ( ((unit or units or school or schools or hospital or hospitals or cluster or clusters or region or regions or ward or wards or practice or practices or community or communities or population or populations or facility or facilities or practitioner or practitioners) n15 random*) ) OR AB ( ((unit or units or school or schools or hospital or hospitals or cluster or clusters or region or regions or ward or wards or practice or practices or community or communities or population or populations or facility or facilities or practitioner or practitioners) n15 random*) )

8. TI ( ((unit or units or school or schools or hospital or hospitals or cluster or clusters or region or regions or ward or wards or practice or practices or community or communities or population or populations or facility or facilities or practitioner or practitioners) n15 interven*) ) OR AB ( ((unit or units or school or schools or hospital or hospitals or cluster or clusters or region or regions or ward or wards or practice or practices or community or communities or population or populations or facility or facilities or practitioner or practitioners) n15 interven*) )

9. TI ( ((group* n1 random*) or (group* n1 interven*)) ) OR AB ( ((group* n1 random*) or (group* n1 interven*)) )

10. S7 or S8 or S9

HUMANS ONLY

11. (MH "Human")

12. (MH "Animals")

13. S12 not S11

COMBINE CONCEPTS

14. S6 and S10

15. S14 not S13

16. Exclude MEDLINE
