# Supplementary material for: The quality of reporting in cluster randomised crossover trials: proposal for reporting items and an assessment of reporting quality
Source: Trials. 2016 Dec 6;17:575. doi: 10.1186/s13063-016-1685-6 (PMC5142135; doi:10.1186/s13063-016-1685-6)
Supplement: Additional file 2: Table S1. — Country where the trial was conducted. Table containing additional demographic data of the included trials in the review. (DOCX 12 kb) [file 13063_2016_1685_MOESM2_ESM.docx]

**Table S1: Country where the trial was conducted**

| **Country** | **n (%)**  **(N = 83)** |
| --- | --- |
| USA | 29 (35%) |
| UK | 10 (12%) |
| The Netherlands | 8 (10%) |
| Canada | 5 (6%) |
| More than 1 country | 3 (4%) |
| Australia | 3 (4%) |
| France | 3 (4%) |
| China | 2 (2%) |
| Denmark | 2 (2%) |
| Germany | 2 (2%) |
| Sweden | 2 (2%) |
| Austria | 1 (1%) |
| Belgium | 1 (1%) |
| Estonia | 1 (1%) |
| Finland | 1 (1%) |
| Greece | 1 (1%) |
| Kenya* | 1 (1%) |
| Pakistan | 1 (1%) |
| South Korea | 1 (1%) |
| South Africa | 1 (1%) |
| Switzerland | 1 (1%) |
| Taiwan | 1 (1%) |
| Tanzania* | 1 (1%) |
| Thailand* | 1 (1%) |
| Zambia* | 1 (1%) |

* Developing countries as classified by the International Monetary Fund, 2015
