# Supplementary material for: The quality of reporting in cluster randomised crossover trials: proposal for reporting items and an assessment of reporting quality
Source: Trials. 2016 Dec 6;17:575. doi: 10.1186/s13063-016-1685-6 (PMC5142135; doi:10.1186/s13063-016-1685-6)
Supplement: Additional file 3: Table S2. — Type of randomised cluster. Table containing additional demographic data of the included trials in the review. (DOCX 12 kb) [file 13063_2016_1685_MOESM3_ESM.docx]

**Table S2: Type of randomised cluster**

| **Randomised cluster type** | **n (%)**  **(N = 83)** |
| --- | --- |
| Hospital or ward | 40 (48%) |
| ICU | 17 |
| Other wards | 23 |
| Individual health care provider | 13 (16%) |
| School or class | 11 (13%) |
| Class or classroom | 7 |
| School | 3 |
| Group of students | 1 |
| Emergency medical team | 6 (7%) |
| Primary care practice | 3 (4%) |
| Individual (mouth, muscles) | 2 (2%) |
| Dementia unit or facility | 2 (2%) |
| Aged care facility | 2 (2%) |
| Community or geographical area | 1 (1%) |
| Household or family group | 1 (1%) |
| Workplace | 1 (1%) |
| Outpatient clinic | 1 (1%) |

ICU: Intensive care unit
